# Researches as to the Amount of Alkaline Reserve in the Blood Serum of the Wounded

**Published:** 1918-04

**Authors:** 


***Researches as to the Amount of Alkaline Reserve in the Blood Serum of the Wounded.*** By EDGARD ZUNZ. Abs. from the *Ambulance de " l'Ocean "*, Fascicule II, Dec. 1917.

The alkaline reserve in the blood plasma is composed of bicarbonates, carbonates, phosphates, and proteins in combination with alkali. The amount of alkali reserved in the serum does not seem to vary with the time of day or with the proximity to meals. Neither is it affected by ordinary wounds or closed fractures unaccompanied by fever or serious infection. Even in open fractures without infection when the tissue damage is not very great, the reserve remains normal.

When the temperature reaches 38° C., $Rp_h$ usually falls to 8.35 in open fractures, but rises to 8.4 or 8.45 or 8.5 as the temperature becomes normal. When there is streptococcic infection of an open fracture a slight degree of acidosis is observed and $Rp_h$ falls to 8.25, rising to normal as the infection passes off. The alkaline reserve shows similar variations in connection with very large wounds accompanied by infection.

In cases of gas gangrene which causes more serious acidosis than streptococcic infection, $Rp_h$ falls sometimes as low as 7.9, 7.8, or 7.7.

In wounds involving dyspnea $Rp_h$ tends to fall. In bronchopneumonia it falls to 7.9, and in fatal cases to 7.7 before death.

Acidosis observed in shock the writer attributes to difficulty in breathing and to symptoms of asphyxia.

He observes that the lowest values of the alkaline reserve tend to coincide with the maximum of infection and the maximum of leucocytes in the blood. When $Rp_h$ falls below 7.8 the prognosis is very doubtful.

